# Clinical features of patients undergoing hemodialysis with COVID‐19

**DOI:** 10.1111/sdi.12928

**Published:** 2020-10-29

**Authors:** Ming Tian, Hua Li, Ting Yan, Yujie Dai, Liping Dong, Honglan Wei, Xiaohong Song, Junwu Dong, Fangxiong Cheng, Wenzhou Li

**Affiliations:** ^1^ Department of Nephrology Wuhan Fourth Hospital Puai Hospital Tongji Medical College Huazhong University of Science and Technology Wuhan P.R. China; ^2^ Department of Nephrology The First People's Hospital of Jiangxia District Wuhan P.R. China; ^3^ Department of Clinical Laboratory Wuhan Fourth Hospital Puai Hospital Tongji Medical College Huazhong University of Science and Technology Wuhan P.R. China; ^4^ Department of Urology Wuhan Fourth Hospital Puai Hospital Tongji Medical College Huazhong University of Science and Technology Wuhan P.R. China

**Keywords:** clinical features, coronavirus disease 2019, hemodialysis, SARS‐CoV‐2

## Abstract

Hemodialysis patients are susceptible to coronavirus disease 2019 (COVID‐19). The aim of this study was to describe the epidemiological, clinical characteristics, and mortality‐related risk factors for those who undergoing hemodialysis with COVID‐19. We conducted a retrospective study. A total of 49 hemodialysis patients with COVID‐19 (Group 1) and 74 uninfected patients (Group 2) were included. For patients in Group 1, we found the median age was 62 years (36‐89 years), 59.3% were male, and the median dialysis vintage was 26 months. Twenty‐eight patients (57%) had three or more comorbidities and two patients (4%) died. The most common symptoms were fever (32.7%) and dry cough (46.9%), while nine patients (18.4%) were asymptomatic. Blood routine tests indicated lymphocytopenia, the proportion of lymphocyte subsets was generally reduced, and chest CT scans showed ground‐glass opacity (45.8%) and patchy shadowing (35.4%). However, these findings were not specific to hemodialysis patients with COVID‐19, and similar manifestations could be found in patients without SARS‐CoV‐2 infection. In conclusion, for hemodialysis patients with COVID‐19, lymphocytopenia and ground‐glass opacities or patchy opacities were common but not specific to them, early active treatment and interventions against nosocomial infection can significantly reduce the mortality and the risk of SARS‐CoV‐2 infection.

## 1 INTRODUCTION

Since December 2019, coronavirus disease (COVID‐19) has spread rapidly in China and worldwide.^1^ The death toll far exceeded that of SARS (774 deaths), MERS (858 deaths), and Ebola (11,323 deaths) summed together.^2^, ^3^ COVID‐19 embodies the characteristics of diverse clinical manifestations, rapid transmission, complications, and poor prognosis among older adults.^4^, ^5^ The estimated basic reproductive number (R0) of severe acute respiratory syndrome corona virus 2 (SARS‐CoV‐2) is approximately 2.68,^6^ which indicates that herd infection may occur in the absence of appropriate prevention and control measures. Apart from strict travel restrictions and home quarantine, effective control measures are lacking.^7^ Essentially, the treatment procedure and setting in the hemodialysis room is completely in contrast to the current isolation policy because the patients are supposed to be relatively crowded and highly mobile. That the end‐stage kidney disease (ESKD) patients need long‐term aggregate hemodialysis appreciably increases the risk of herd infection and disease transmission, making the prevention and control of infectious diseases more challenging than that in the general population.^8^ Still, there are limited epidemiological data on hemodialysis patients with COVID‐19 during the outbreak of COVID‐19 caused by SARS‐CoV‐2. An early study showed that 37 of the 230 patients enrolled were infected with SARS‐CoV‐2, among whom six died (crude mortality rate: 16.2%).^9^ This suggests that it is imperative to take effective protective interventions to contain the spread of SARS‐CoV‐2 in dialysis units. In the present study, we conducted a retrospective study on the epidemiological characteristics of such special population to foster clinicians’ better understanding.

## 2 MATERIALS AND METHODS

### 2.1 Participants

We retrospectively reviewed patients undergoing hemodialysis with suspected or confirmed COVID‐19 who were admitted or transferred to the Hemodialysis Center of Wuhan Fourth Hospital from other hospitals between February 3 and March 22, 2020, and conducted an analysis of them. Patients with incomplete baseline data and, acute kidney injury receiving hemodialysis were excluded.

### 2.2 Methods

We collected baseline data of patients, including demographics, primary kidney disease, dialysis vintage, vascular access, and comorbidities including diabetes, hypertension, kidney transplantation, cerebrovascular disease, cardiovascular disease, systemic lupus erythematosus. We also collected the laboratory and radiological findings of patients during the outbreak or during the disease, including blood routine, myocardial enzymes, hepatic, and renal function, electrolytes, ferritin, parathyroid hormone, prothrombin time (PT), activated partial thromboplastin time (APTT), D‐dimer, high‐sensitive C‐reactive protein (hs‐CRP), lymphocyte subsets, chest CT scan, SARS‐CoV‐2 nucleic acid results of nasopharyngeal swabs, and SARS‐CoV‐2 specific antibodies (colloidal gold method, reagent No. 20203400177, from Innovita (Tangshan) Biotech Co., Ltd.).^10^ Blood specimens for biochemical tests were collected from the vascular access before midweek hemodialysis sessions. They were collected 7:00‐8:00 h before morning sessions and 11:00‐12:00 h before midday sessions. The patients were grouped into Group 1 (patients undergoing hemodialysis with COVID‐19) and Group 2 (uninfected with COVID‐19).

### 2.3 Criteria for diagnosis and clinical prognosis

All patients are in compliance with the Health Committee of the people's Republic of China about the criteria for COVID‐19 diagnosis and treatment plan (trial version 7) at http://www.nhc.gov.cn/yzygj/s7652m/202003/a31191442e29474b98bfed5579d5af95.shtml.

### 2.4 Endpoint of the study

All patients were followed up (Tracking by phone and electronic medical records) until the end event (all‐cause mortality), loss to follow‐up, or study deadline on April 11, 2020.

### 2.5 Statistical analysis

Continuous variables were described by median, minimum, and maximum values, whereas categorical variables were described by counts and percentages. The differences between the two Groups were compared using Mann–Whitney U rank‐sum test. A value of *p* < 0.05 was considered statistically significant. All statistical results were calculated using R software version 3.6.3. Graphics were made using Origin version 9.1 and Photoshop version 6.0.

## 3 RESULTS

### 3.1 Epidemiological characteristics

**FIGURE 1:**
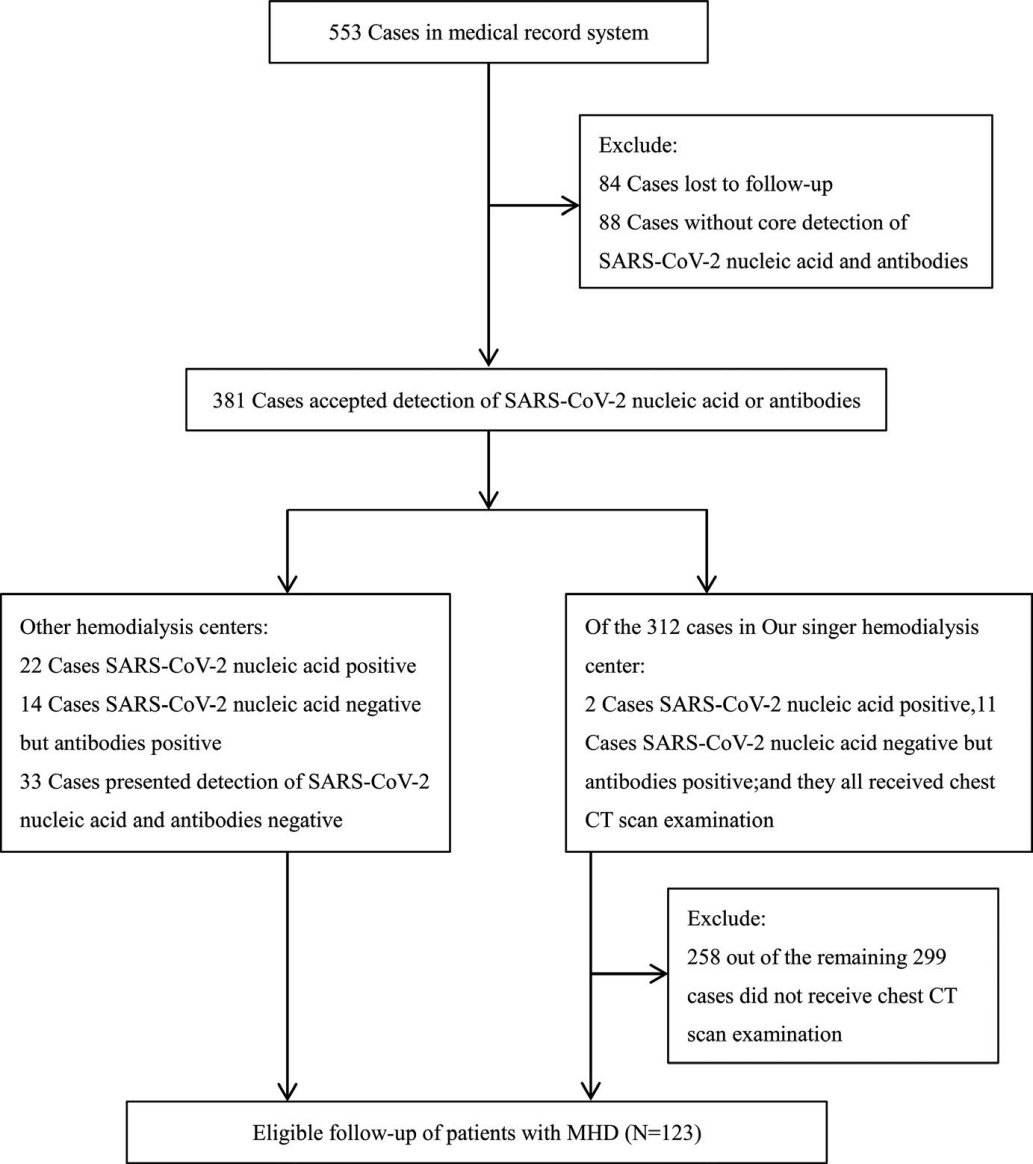
The flow chart of patient recruitment. After excluding 430 patients, the final sample size of 123 participants was enrolled.

At that time, Wuhan was at the epicenter of the epidemic in China. The hemodialysis centers of Wuhan Fourth Hospital in Gutian District were one of the first designated hospitals to care for hemodialysis patients with confirmed or suspected COVID‐19. There were two hemodialysis units, one was in Building 1 with negative‐pressure isolation ward, and 56 hemodialysis machines for patients with suspected or confirmed COVID‐19 infection who were transferred from other hospitals, the other was in building 3 with 72 hemodialysis machines for uninfected hemodialysis patients. A total of 553 patients on maintenance hemodialysis were enrolled in this study, and 123 cases (Figure 1) were eventually selected according to the inclusion and exclusion criteria.

The demographic and clinical characteristics of the patients are shown in Table 1.

**TABLE 1:** Demographic and Clinical Characteristics of the Patients at Baseline.

Characteristic	Total	Group 1	Group 2	*p* value
	N = 123	N = 49	N = 74	
Age, Median (range)‐years	62 (23‐89)	62 (36‐89)	62.5 (23‐88)	0.741
Male‐No., %	73 (59.3%)	30 (61.2%)	43 (58.1%)	0.73
BMI‐kg/m^2^, median (range)‐years	21.6 (13.2‐32)	21.9 (16.2‐27.8)	21.5 (13.2‐32)	0.153
Dialysis vintage, median (range)‐month	45 (0.5‐240)	26 (0.5‐218)	60 (1‐240)	0.107
Vascular access‐no., %				
Tunneled cuffed catheter	37 (30.1%)	15 (30.6%)	22 (29.7%)	0.917
AvF	86 (69.9%)	34 (69.4%)	52 (70.3%)	
Dialysis frequency‐no., %				
Twice per week	46 (37.4)	17 (34.7%)	29 (39.2%)	0.013
Five times in 2 weeks	17 (13.8%)	2 (4.1%)	15 (20.3%)	
Three times per week	60 (48.8%)	30 (61.2%)	30 (40.5%)	
Primary renal disease‐no., %				
Glomerulonephritis	52 (42.3%)	18 (36.7%)	34 (45.9%)	0.486
Diabetic nephropathy	36 (29.3%)	15 (30.6%)	21 (28.4%)	
Hypertensive kidney disease	18 (14.6%)	7 (14.3%)	11 (14.9%)	
Polycystic kidney disease	7 (5.7%)	3 (6.1%)	4 (5.4%)	
Lupus nephritis	2 (1.6%)	2 (4.1%)	0 (0.0%)	
Obstructive nephropathy	4 (3.3%)	1 (2.0%)	3 (4.1%)	
Drug‐induced kidney damage	1 (0.8%)	1 (2.0%)	0 (0.0%)	
Gouty nephropathy	3 (2.4%)	2 (4.1%)	1 (1.4%)	
Kidney transplant‐no., %	1 (0.8%)	0 (0.0%)	1 (1.4%)	0.414
Hypertension‐no., %	122 (99.2%)	49 (100.0%)	73 (98.6%)	0.414
Diabetes‐no., %	39 (31.7%)	17 (34.7%)	22 (29.7%)	0.562
Stroke‐no., %	18 (14.6%)	5 (10.2%)	13 (17.6%)	0.306
Coronary heart disease‐no., %	27 (22%)	12 (24.5%)	15 (20.3%)	0.58
Signs and symptoms at admission (Group 1) or during the outbreak (Group 2) ‐no., %				
Asymptomatic	56 (45.5%)	9 (18.4%)	47 (63.5%)	<0.001
Fever	22 (17.9%)	16 (32.7%)	6 (8.1%)	<0.001
Cough	40 (32.5%)	23 (46.9%)	17 (23.0%)	0.005
Sputum production	11 (8.9%)	7 (14.3%)	4 (5.4%)	0.113
Rhinorrhea	1 (0.8%)	0 (0.0%)	1 (3.1%)	0.395
Sore throat	1 (0.8%)	0 (0.0%)	1 (1.4%)	0.414
Shortness of breath	12 (9.8%)	7 (14.3%)	5 (6.8%)	0.218
Anorexia	8 (6.5%)	6 (12.2%)	2 (2.7%)	0.058
Nausea and vomiting	4 (3.3%)	4 (8.2%)	0 (0.0%)	0.023
Diarrhea	4 (3.3%)	4 (8.2%)	0 (0.0%)	0.023
Fatigue	16 (13%)	13 (26.5%)	3 (4.1%)	<0.001
Muscle ache	1 (0.8%)	1 (2.0%)	0 (0.0%)	0.398

Abbreviations: AvF, arteriovenous fistula; BMI, Body Mass Index.

**FIGURE 2:**
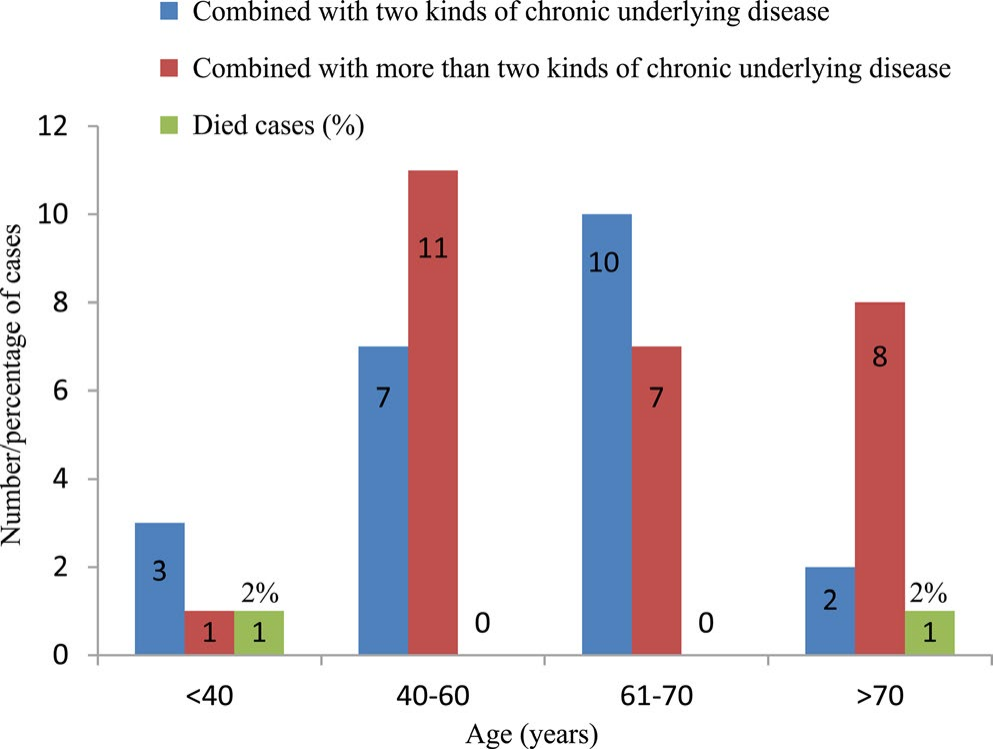
Number/percentage of cases of age distribution, cumulative comorbidities and death rate with COVID‐19 in Group 1. Comorbidities including diabetes, hypertension, kidney transplantation, cerebrovascular disease, cardiovascular disease, and systemic lupus erythematosus.

There was no significant difference in epidemiological data between Group 1 and Group 2. In Group 1, the median age of patients was 62 years (36‐89 years). Four cases were under 40 years (8.2%), 18 cases were between 40 and 60 years (36.7%), 17 cases were between 61 and 70 years (34.7%), and 10 cases were above 70 years (20.4%). Most of the patients were male (61.2%). The longest dialysis vintage was 218 months, and the median dialysis vintage was 26 months. Overall, 69.4% of patients had arteriovenous fistula as vascular access. The frequency of dialysis was three times a week in most patients and five times in 2 weeks in very few patients. The primary diseases that caused ESKD were diverse, among which primary glomerulonephritis and diabetes predominated, accounting for 36.7% and 30.6%, respectively. Moreover, we found that more than 50% of the patients had three or more chronic underlying diseases, and the proportion increased with age (Figure 2).

### 3.2 Clinical symptoms and signs

**FIGURE 3:**
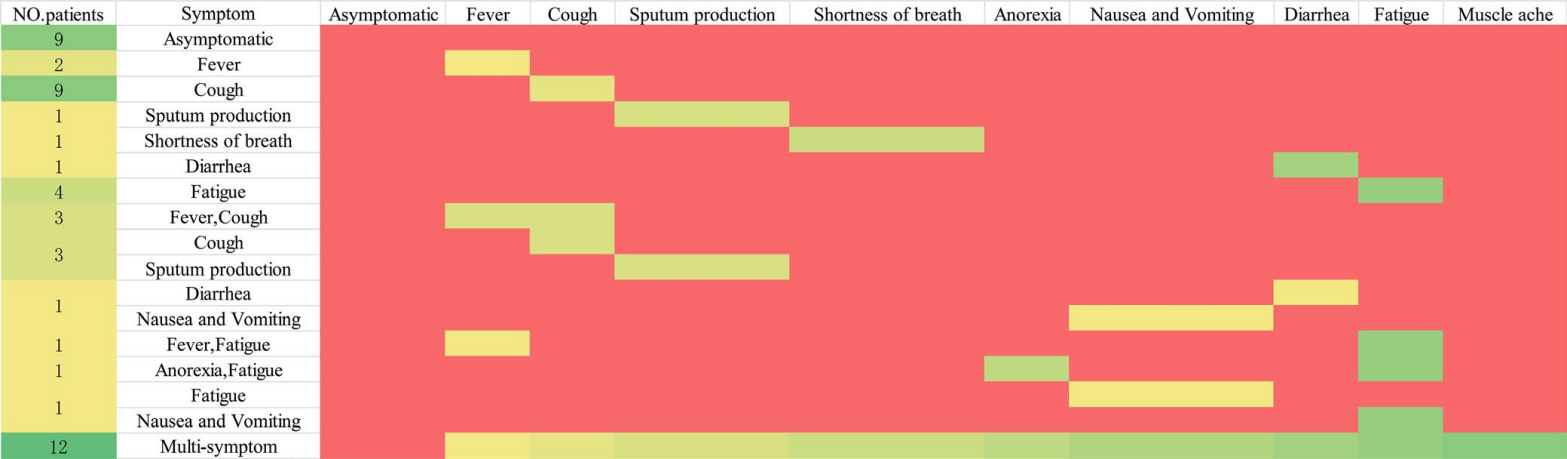
Horizontal representation of symptoms of patients. No. = number, red indicates no corresponding symptoms, other colors indicate corresponding symptoms.

To better understand the clinical symptoms of hemodialysis patients with COVID‐19, we considered Group 2 patients who were uninfected during the outbreak as the control Group. The results for Group 1 showed that the symptoms of the patients included asymptomatic infection, fever, dry cough, expectoration, chest tightness and shortness of breath, anorexia, nausea and vomiting, diarrhea, fatigue, and muscle soreness. Nine cases (18.4%) were asymptomatic. Among them, two patients were found to have significantly increased hs‐CRP or significantly decreased lymphocytes in a routine blood test, and further screening revealed infection with COVID‐19. In the other seven patients, chest CT scan revealed viral pneumonia‐like manifestations, and further tests of SARS‐CoV‐2 nucleic acid and specific antibodies confirmed infection. Of all the patients, 16 (32.7%) developed fever, 23 (46.9%) had cough, and 13 (26.5%) patients had fatigue, whereas other symptoms such as chest tightness and shortness of breath, nausea and vomiting, and muscle soreness were rare. Twelve patients (24.5%) complained of three or more clinical symptoms at the same time (Table 1 and Figure 3).

### 3.3 Laboratory results, radiographic findings, and clinical outcomes

**FIGURE 4:**
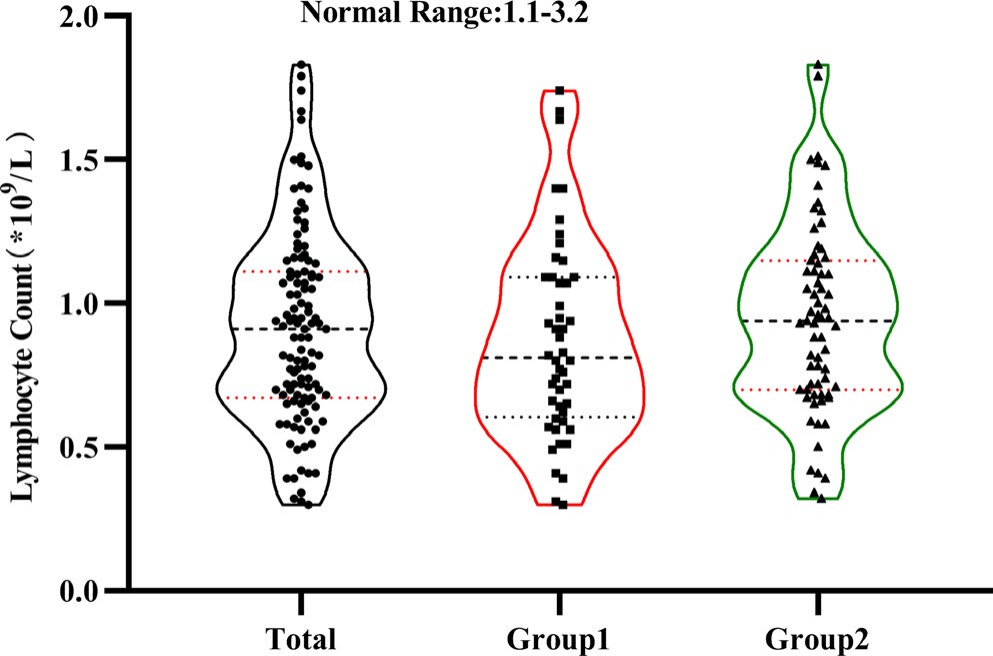
Display the distribution of lymphocytes count in the two Groups. Black dots indicate the actual test value of the patient. Dotted lines represent the distribution of quartiles.

**FIGURE 5:**
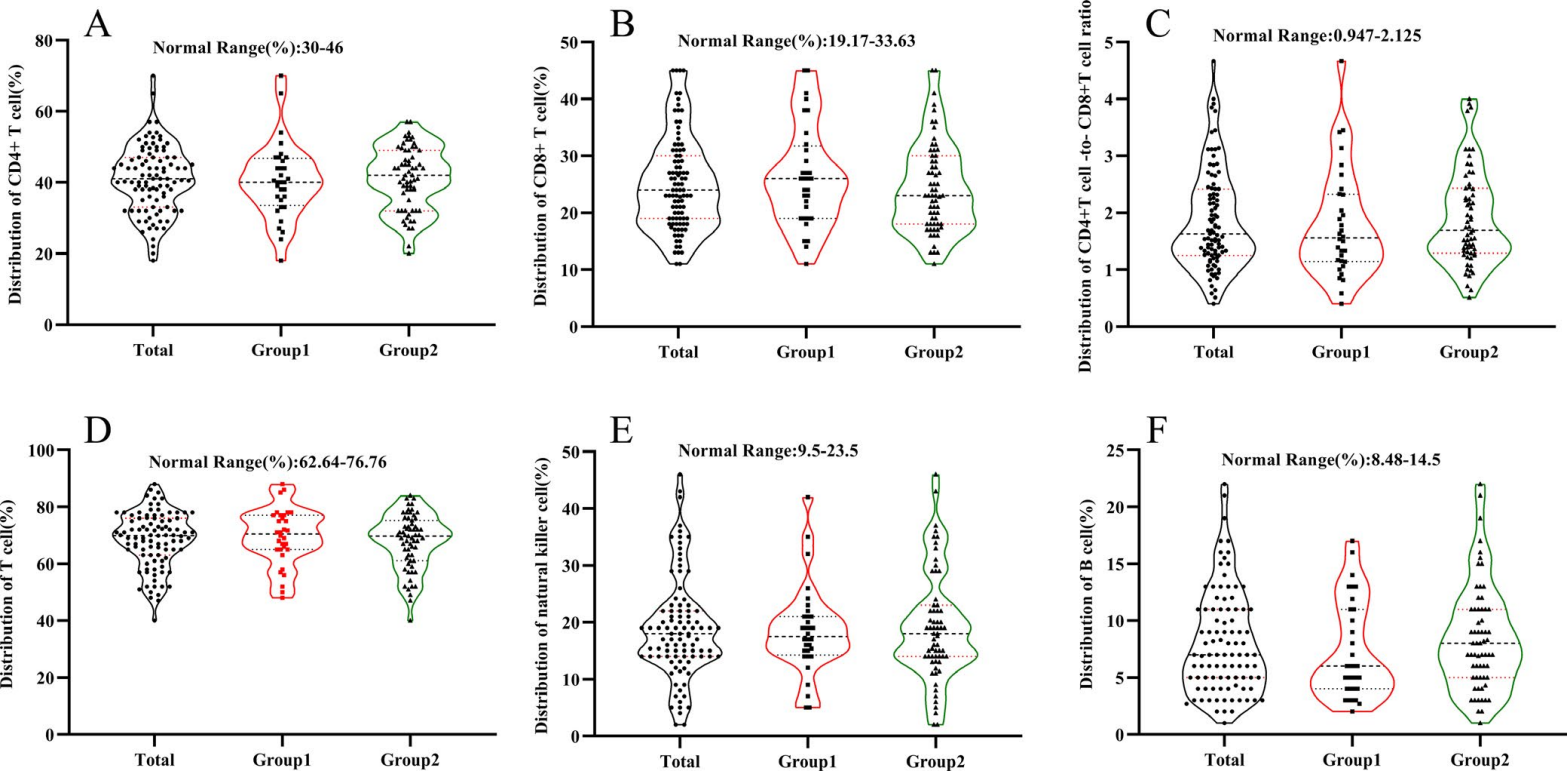
Distribution percentage of CD4+ T cell (A), CD8+ T cell (B), CD4+ T cell ‐to‐ CD8+ T cell ratio (C), T cell (D), natural killer cell (E) and B cell (F) in the two Groups. Black or red dots indicate the actual test value of the patient.

For laboratory tests, we perceived no appreciable differences in white blood cell count, neutrophil count, lymphocyte count, myocardial enzymes, renal function, or coagulation function between the two Groups. Lymphocytopenia occurred regardless of whether the patient was infected with COVID‐19. To better understand whether there was a difference in the distribution of lymphocyte count between the two Groups, we conducted quartile grouping in Figure 4. Furthermore, the results showed that the median hs‐CRP and ferritin in Group 1 were appreciably higher than that in Group 2. Although D‐dimer levels in Group 1 were higher than that in Group 2, because the vast majority of patients use heparin for dialysis, the difference in coagulation function between the two Groups was not easy to identify. To determine whether there were changes in lymphocyte subsets between the two Groups, we examined the two Groups of patients and found that there were no significant changes in the proportions of CD8 + T cells, CD4 + T cells, Natural killer cells, and B cells. Moreover, there was no significant difference in CD4‐to‐CD8 ratio between the two Groups in Figure 5 and Table 2.

**TABLE 2:** Laboratory results, radiographic findings, clinical outcome among different Groups.

Characteristic Median (range) or No., %	Normal range	Total	Group 1	Group 2	*p* value
		N = 123	N = 49	N = 74	
WBC Count, ×109/L	3.5‐9.5	5.12(2.02‐14.27)	4.84(2.02‐12.4)	5.56 (2.8‐14.27)	0.03
Neutrophil count (×109/L)	1.8‐6.3	3.61 (1‐12.32)	3.33 (1‐9.97)	3.93 (1.48‐12.32)	0.043
Lymphocyte count (×109/L)	1.1‐3.2	0.91 (0.3‐1.83)	0.81 (0.3‐1.74)	0.94 (0.32‐1.83)	0.159
Platelet Count, ×109/L	125‐350	164 (39‐867)	165 (39‐416)	164 (61‐867)	0.222
Hemoglobin, g/L	115‐150	95 (48‐163)	90 (48‐163)	99 (51‐134)	0.161
Albumin, g/L	40‐55	37.3 (26.9‐51.8)	34.4 (26.9‐46.7)	38.7 (29.7‐51.8)	0.001
ALT, U/L	9‐50	11 (1‐80)	11 (3‐26)	12 (1‐80)	0.562
AST, U/L	13‐40	14 (4‐56)	15 (5‐56)	12 (4‐44)	0.02
LDH, U/L	120‐250	199 (95‐382)	212 (112‐382)	192 (95‐333)	0.105
CK, U/L	50‐310	70 (15‐801)	71 (15‐253)	68 (24‐801)	0.576
CK‐MB, U/L	0‐24	8 (1‐40)	8 (1‐40)	8 (2‐21)	0.818
Blood glucose, mmol/L	3.9‐6.1	6.16 (2.29‐29.86)	5.24 (2.29‐17.7)	7.36 (3.8‐29.86)	0.001
BUN, mmol/L	3.1‐8.8	26.2 (6.04‐62.14)	24.63 (6.04‐62.14)	26.84 (6.5‐48.11)	0.211
Creatinine, μmol/L	41‐81	942.8 (286.9‐2513)	890.3 (286.9‐2513)	956 (421‐1899)	0.255
PT, s	9‐13	11.9 (9.3‐16.6)	11.7 (9.3‐15)	12.2 (9.9‐16.6)	0.137
APTT, s	20‐40	30.7 (19.2‐70.2)	30.9 (19.2‐52.9)	30.1 (20.1‐70.2)	0.757
D‐DIMER, ng/ml	<0.5	0.85 (0.11‐14.17)	1.4 (0.22‐10.84)	0.65 (0.11‐14.17)	0.001
PCT, ng/ml	0‐0.05	0.56 (0.04‐3.86)	0.57 (0.04‐3.86)	0.55 (0.19‐3.18)	0.347
hs‐CRP, mg/L	0‐10	11.2 (0.61‐142.6)	21.75 (0.61‐142.6)	4.6 (1.2‐119)	0.007
Calcium, mmol/L	2.11‐2.52	2.22 (1.42‐2.63)	2.13 (1.42‐2.56)	2.27 (1.82‐2.63)	0.007
Phosphorus, mmol/L	0.85‐1.51	1.87 (0.91‐3.97)	1.81 (0.91‐3.97)	1.94 (1.04‐2.9)	0.41
Ferritin, ng/ml	13‐400	276 (22.8‐1651)	384.5 (56.3‐1651)	243.2 (22.8‐1651)	0.017
PTH, ng/L	15‐65	402.6 (10.4‐2685)	460.2 (69.8‐1162.8)	377.8 (10.4‐2685)	0.92
Lymphocyte subsets					
T cells, %	62.64‐76.76	69.9 (40‐88)	70.5 (48‐88)	69.7 (40‐84)	0.589
CD8+ T cells, %	19.17‐33.63	24 (11‐45)	26 (11‐45)	23 (11‐45)	0.307
CD4+ T cells, %	30‐46	41 (18‐70)	40 (18‐70)	42 (20‐57)	0.466
Natural killer cells, %	9.5‐23.5	18 (2‐46)	17.5 (5‐42)	18 (2‐46)	0.931
B cells, %	8.48‐14.5	7 (1‐22)	6 (2‐17)	8 (1‐22)	0.214
CD4/CD8 ratio	0.947‐2.125	1.63 (0.4‐4.67)	1.56 (0.4‐4.67)	1.69 (0.51‐4.0)	0.378
Chest CT scan findings‐No., %					
Normal		12	2 (4.2%)	10 (14.1%)	<0.001
Unilateral pneumonia		16	0 (0.0%)	16 (22.5%)	
Bilateral pneumonia		21	7 (14.6%)	14 (19.7%)	
Ground‐glass opacity		39	22 (45.8%)	17 (23.9%)	
Unilateral or bilateral patchy shadowing		31	17 (35.4%)	14 (19.7%)	
Clinical outcome in Group 1					
Died			2 (4.1%)		—
Discharged			43 (87.7%)		
Remained in hospital			4 (8.2%)		

Abbreviations: APTT, activated partial thromboplastin time; BUN, blood urea nitrogen; CK, creatine kinase; CK‐MB, creatinine kinase–myocardial band; hs‐CRP, hypersensitive c‐reactive protein; LDH, lactate dehydrogenase; PCT, procalcitonin; PT, prothrombin time; PTH, parathyroid hormone; WBC, white blood cell.

**FIGURE 6:**
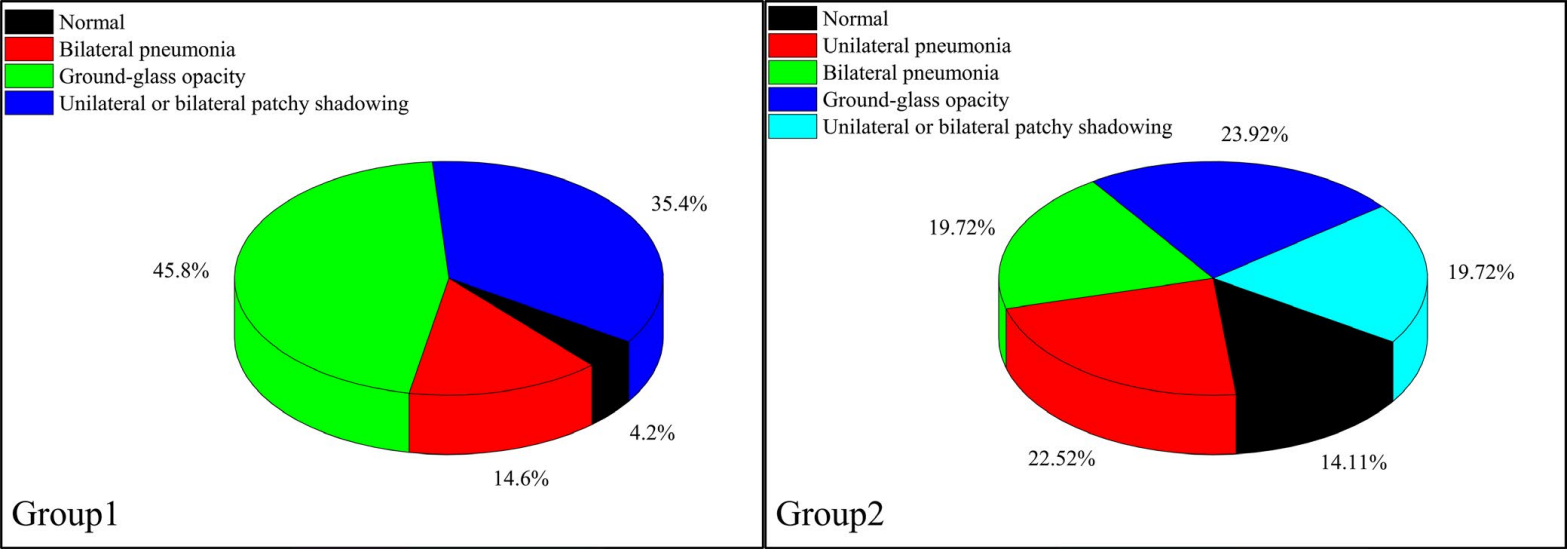
Proportion of different imaging manifestations in chest CT scan within Group 1 and Group 2.

Collecting 48 chest CT scan results from Group 1, we found that patients undergoing hemodialysis with COVID‐19 often had characteristic changes, mainly ground‐glass opacity (45.8%) and patchy shadowing of the lungs (35.4%). It was all known that hemodialysis patients appreciably increased the risk of herd infection and disease transmission,^11^ we suggested every patient receive SARS‐CoV‐2 nucleic acid, chest CT and other examinations so as to find out the potential infected person as much as possible. We chose non‐infected patients with complete examination as the control Group, and similar findings were found in Group 2, but in lower proportions. A small number of patients showed pneumonia changes in bilateral lungs (14.6%) in Group 1. It is worth noting that two patients were diagnosed with COVID‐19, but their chest CT findings were completely normal. By the end of follow‐up, two patients (4.0%) died from COVID‐19 (aged 37 and 71 years, respectively), four cases (8.2%) were still hospitalized, and 43 cases (87.7%) were discharged (Table 2 and Figures 2 and 6).

## 4 DISCUSSION

Our study comprehensively described the key differences in clinical characteristics between patients undergoing hemodialysis with or without COVID‐19. Asymptomatic SARS‐CoV‐2 infections reached 18.4%, and patients with fever merely accounted for 32.7% of the infected cases. Additionally, hs‐CRP and serum ferritin levels were significantly higher in the infected Group than those in the uninfected Group, and ground‐glass opacity and patchy shadowing in the lungs revealed by chest CT scans were more common in the infected Group. Despite the fact that there were many underlying diseases in hemodialysis patients, the mortality rate was not appreciably higher than that of patients not on hemodialysis because of early identification and isolation and providing supportive care. Our study suggested a COVID‐19 infection rate of 4.2% (13/312), which implied that reasonable counseling, education, and management could effectively reduce the risk of infection in spite of a crowded patient population, high mobility, and high risk of infection in the hemodialysis room.

Previous studies have suggested that almost all populations are susceptible to SARS‐COV‐2. Among the 49 patients in the present study, 44.9% were under 60 years, and 55.1% were above 60 years. However, it remains unclear whether the advanced age is a risk factor for susceptibility to COVID‐19.^12^ Patients undergoing hemodialysis with COVID‐19 have many clinical manifestations, among which fever, dry cough, and fatigue and discomfort are dominant, whereas diarrhea, myalgia, anorexia, and shortness of breath are rare.^13^, ^14^, ^15^ Similar characteristics were found in our study. A large‐scale epidemiological study in China suggested that 1.2% of patients had asymptomatic infection and 43.8% had fever at the early stage of the disease,^16^, ^17^ whereas in the present study 18.4% of patients had asymptomatic and 32.7% had fever. However, according to the recommendations of the World Health Organization (WHO),^18^ patients need to have fever and at least one respiratory symptom to be considered a suspected case of COVID‐19 that may have contributed to patients with early COVID‐19 to be underdiagnosed and resulted in increased transmission of the virus. Lymphocytopenia and ground‐glass opacity are typical findings in infected patients,^17^ but little is known about the lymphocyte subsets and immune responses in COVID‐19 patients. We found that ESKD patients had some of their own characteristics compared with non‐dialysis patients infected with COVID‐19,^19^ and that both Groups of patients had lymphocytopenia. There was no significant difference in lymphocyte count and lymphocyte subsets between the two Groups that may be related to extensive damage to lymphocyte and granulocyte functions caused by ESKD status, whose responses to SARS‐CoV‐2 infection may be changed by an abnormal immune system.^20^ Early studies have shown that COVID‐19 patients with comorbidities tend to have a poor prognosis.^21^, ^22^, ^23^ In our study, more than 50% were above 60 years and had multiple diseases in Group 1, but they did not contribute to a significant increase in mortality. The mortality in the present study was higher than the crude mortality rate of COVID‐19 patients in China but lower than the recently reported crude mortality rate of Italian patients (4% vs 2.3% vs, 7.2%).^14^, ^24^ The reason for the low mortality rate of dialysis patients may be that compared with non‐dialysis patients, dialysis patients can make better use of nearby medical resources due to frequent encounters with medical care and be treated at the early stage of the disease, so that medical runs are less likely to cause treatment delays.

Effective infection control intervention is the sole way to prevent the spread of SARS‐CoV‐2 so far,^25^ at the core of which during the outbreak is early identification and isolation and providing supportive care. Unlike ordinary patients, those who undergoing hemodialysis visit hospitals frequently and need to go to designated hospitals for hemodialysis two to three times a week. Due to a dense population and high mobility in the dialysis room, patients repeatedly going back and forth between the family and the hospital caused significantly increased infection and transmission of SARS‐CoV‐2. There were more than 400 patients with maintenance hemodialysis in the Hemodialysis Center of Wuhan Fourth Hospital. To effectively reduce the risk of herd infection in these patients, we undertook many measures to keep the infection risk below 5%. In summary, many measures can be used for reference: highlight the importance of prevention, screening, isolation, testing, and curing routine at all levels—dialysis unit, community/house, transportation, family members as key to preventing the spread even in a vulnerable population being treated in a relatively closed space. In order to reduce cross‐infection between medical staff and patients, as for medical staff, we regularly organized online learning of related epidemiology, prevention, and control knowledge about COVID‐19. Various meetings were held by video conference as much as possible, so as to reduce crowd gathering. Staffs needed to monitor their symptoms every day, family members and other persons suspected or confirmed to be infected with COVID‐19 and in close contact with them should also report to the department leader in time. We asked everyone to wear protective clothing, Cap, gloves, goggles, and N95 face mask when on duty. Also we recommend staffs eat at different times and avoid eating together. Tried to talk less when dining. Wash hands for disinfection frequently.

There are several limitations to the present study. First, this study was a retrospective study with a small sample size; further large‐scale cohort studies are needed to confirm the findings. Second, it remains unclear whether there is superinfection of bacteria and other viruses in patients undergoing hemodialysis with COVID‐19 that affects the immune response. Third, the mortality rate reported in this study should not be confused with the true case fatality rate, because four patients were still hospitalized as of the follow‐up date although their condition was not particularly serious. Fourth, the follow‐up time of this study is short. We are not clear about the long‐term effect of COVID‐19 on hemodialysis patients, and we need longer observation in the later work.

In conclusion, we found that hemodialysis patients with COVID‐19 had various symptoms. Lymphocytopenia could occur irrespective of whether the patient was infected with COVID‐19. Chest CT scans showed ground‐glass opacity or patchy shadowing in the majority of patients, but they were not specific to patients with COVID‐19 and occurred in patients undergoing hemodialysis without COVID‐19 in a lower proportion. This makes the diagnosis of patients undergoing hemodialysis with COVID‐19 more complex and difficult, and the tests of SARS‐CoV‐2 nucleic acid and specific antibodies should be completed simultaneously. For hemodialysis patients with COVID‐19, early active treatment, health education, and interventions against nosocomial infection can significantly reduce the mortality and the risk of SARS‐CoV‐2 infection.

## CONFLICT OF INTEREST

All authors have declared no conflict of interest.

## AUTHORS' CONTRIBUTIONS

J.W.D., F.X.C. and W.Z.L. conceived and supervised the project. M.T., H.L. wrote the manuscript. L.P.D. and H.L.W. assisted in interpretation of data and writing of the manuscript. T.Y., L.P.D., Y.J.D, H.L.W., and M.T. collected the data. M.T, H.L., J.W.D. and F.X.C. performed all statistical analyses and graphing. All authors discussed the results, read and approved the final manuscript.

## ETHICAL APPROVAL

All procedures performed in accordance with the ethical standards of the institutional research committee at which the studies were conducted (IRB approval number KY 2020‐013‐01 at the ethics committee of Wuhan Fourth Hospital, and the study conformed to the provisions of the Declaration of Helsinki.
